# Establishment of a preclinical ovine screening model for the investigation of bone tissue engineering strategies in cancellous and cortical bone defects

**DOI:** 10.1186/s12891-016-0964-4

**Published:** 2016-03-01

**Authors:** Anne-Marie Pobloth, Kenneth A. Johnson, Hanna Schell, Nicolai Kolarczik, Dag Wulsten, Georg N. Duda, Katharina Schmidt-Bleek

**Affiliations:** Julius Wolff Institute and Center for Musculoskeletal Surgery, Charité – Universitätsmedizin Berlin, Augustenburger Platz 1, D-13353 Berlin, Germany; Berlin-Brandenburg Center for Regenerative Therapies, Charité – Universitätsmedizin Berlin, Augustenburger Platz 1, D-13353 Berlin, Germany; Faculty of Veterinary Science, University of Sydney, Sydney, 2006 NSW Australia

**Keywords:** Large animal model, Drill hole model, Cortical and cancellous bone defect regeneration

## Abstract

**Background:**

New tissue engineering strategies for bone regeneration need to be investigated in a relevant preclinical large animal model before making the translation into human patients. Therefore, our interdisciplinary group established a simplified large animal screening model for intramembranous bone defect regeneration in cancellous and cortical bone.

**Methods:**

Related to a well-established model of cancellous drill hole defect regeneration in sheep, both the proximal and distal epimetaphyseal regions of the femur and the humerus were used bilaterally for eight drill hole cancellous defects (Ø 6 mm, 15 mm depth). Several improvements of the surgical procedure and equipment for an easier harvest of samples were invented. For the inclusion of cortical defect regeneration, a total of eight unicortical diaphyseal drill holes (6 mm Ø) were placed in the proximal-lateral and distal-medial parts of the metacarpal (MC) and metatarsal (MT) diaphyseal bone bilaterally. Acting moments within a normal gait cycle in the musculoskeletal lower limb model were compared with the results of the biomechanical in vitro torsion test until failure to ensure a low accidental fracture risk of utilized bones (ANOVA, *p* < 0.05). The model was tested in vivo, using thirteen adult, female, black-face sheep (Ø 66 kg; ± 5 kg; age ≥ 2.5 years). In a two-step surgical procedure 16 drill holes were performed for the investigation of two different time points within one animal. Defects were left empty, augmented with autologous cancellous bone or soft bone graft substitutes.

**Results:**

The in vitro tests confirmed this model a high comparability between drilled MC and MT bones and a high safety margin until fracture. The exclusion of one animal from the in vivo study, due to a spiral fracture of the left MC bone led to a tolerable failure rate of 8 %.

**Conclusions:**

As a screening tool, promising biomaterials can be tested in this cancellous and cortical bone defect model prior to the application in a more complex treatment site.

## Background

Although bone possesses a good healing capacity, complications during bone defect regeneration are still a significant clinical concern. Various aspects such as defect size, defect localization, biomechanical conditions, infections, and different underlying diseases affect the healing situation [1, 2]. To promote endogenous bone defect regeneration several strategies are under investigation, e.g. the use of growth factors or scaffold materials for bone tissue engineering [3–5]. A general understanding of these novel therapeutical strategies can be achieved in in vitro cell cultures and in small animal models, but these models are somewhat limited. Feasibility of upsizing promising scaffold solutions to a relevant defect size and adaptation to certain bone structures are reasons for the need of a large animal model before a translation into human patients can be considered. Bone defect regenerative mechanisms, such as direct intramembranous ossification or endochondral ossification, are dependent on the involved bone structure and bone defect size [6]. While larger, diaphyseal bone fractures heal via endochondral bone regeneration, small diaphyseal defects and epimetaphyseal defects affecting the cancellous bone regenerate by direct intramembraneous ossification [7–11]. Additionally, diaphyseal bone essentially heals through periosteal callus formation, while epimetaphyseal bone usually heals endosteally with micro-callus and only minimal periosteal callus formation [8, 9, 12, 13]. New tissue engineering strategies should support the different healing mechanisms involved under consideration of these different requirements. Therefore, a suitable animal model needs to be carefully considered. Several small and large animal models have been established using different defect sizes, critical and non-critical, affecting either the epimetaphyseal or diaphyseal part of long bones [2–5, 14]. However, a validated large animal model offering the possibility to test bone tissue engineering strategies in both cancellous and cortical bone with an adequate sample size is lacking. In this article we show the approach of our multidisciplinary research team to establish a simplified large animal screening model, which allows the analysis of promising biomaterials in both epimetaphyseal and diaphyseal bone simultaneously by using defects of adequate dimension, and at different healing time points, resulting in a reduction of the number of experimental animals.

## Methods

**Fig. 1:**
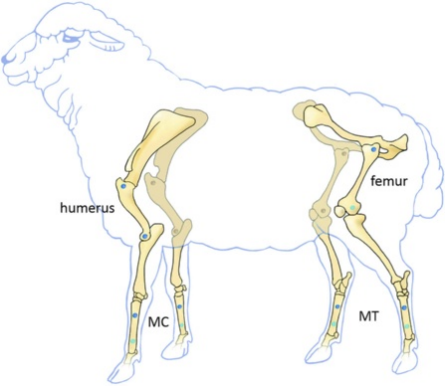
Localization of the drill hole defects within the sheep skeleton (proximal and distal femur and humerus, metacarpal bones = MC, metatarsal bones = MT). Drill holes, which were performed via a lateral approach are marked in dark blue while drill holes performed by a medial approch are marked in light blue (distal femur, distal metacarpal and metatarsal bones). Surgeries on the right legs were performed first followed by a second surgery on the left legs 6 weeks later. Sample harvesting followed 9 weeks after the first surgery and 3 weeks after the second surgery for the investigation of an early and an advanced healing time point

In this new drill hole defect model cancellous and cortical bone defects were investigated in each animal at two observation time points (three and nine weeks). The hitherto established cancellous, epimetaphyseal 8 mm drill hole defect model in Swiss alpine sheep was adapted to the black-faced sheep and modified [15–19]. Eight smaller cancellous drill holes of 6 mm diameter were created for comparison with 6 mm diaphyseal drill holes. Instead of using the lateral femoral condyle, the medial femoral condyle was utilized for one of the cancellous bone holes. To investigate two healing time points in one animal, surgery on the right legs was performed first, followed six weeks later by a second surgery on the left legs of the animal. In addition to the epimetaphyseal drill holes, eight unicortical drill holes were placed in the metacarpal and metatarsal bones for the investigation of cortical bone healing (Fig. 1). Animals were sacrificed nine weeks after the first surgery allowing the investigation of bone defect healing time points of three (left side) and nine (right side) weeks. Thus, for each time point evaluation of four cancellous and four cortical defects were possible.

A drill hole of 6 mm diameter was performed to produce a bone defect size below the recommended 50 % of the corresponding bone diameter (43 – 50 % metacarpal bone; 35–40 % metatarsal bone) for a low fracture risk and an adequate bone defect size for thorough investigations of bone forming biomaterials [20].

### Biomechanical testing

A biomechanical validation of the metacarpal and metatarsal bones used in this model was performed by a torsion test until failure of native bones as well as drilled bones. The native metacarpal and metatarsal bones were compared in this test for a general characterization of the utilized bones. The 6 mm defect size was investigated in the metacarpal and metatarsal bones to confirm that these two drill holes per bone did not result in a stress-riser and bone failure under physiological strains. The drill holes were positioned in the same location as during surgery. Four paired metacarpal and metatarsal bones harvested from four earlier sacrificed adult sheep were used. Eight native bones and the corresponding contralateral bones were each prepared with 6 mm diameter proximal-lateral and distal-medial unicortical drill holes. In order to stabilize the later bone-embedding material compound short k-wires were driven perpendicular through the bone ends. All bones were aligned vertically using a cross-line laser. Afterwards, two centimeters of the bone ends were embedded in the two component plastic Memecryl® (Bauer Handels GmbH, Fehraltdorf, Switzerland) in custom-made test pots. A load to failure test was performed in the Zwick testing machine (Zwick Roell AG, Z010, Ulm, Germany). The test protocol involved an axial pre-load of 0.5 N followed by an axial rotation at a rate of 10 ° / min until failure of the tested structure occurred. Applied rotation and resulting torque were acquired at 10 Hz with the software testExpert® II (Zwick Roell AG, Z010, Ulm, Germany). Subsequent data analysis in Matlab 7.7 (The MathWorks GmbH, Ismaning, Germany) resulted in the maximum torsional moment until failure (Mmax [Nm]), the angle at Mmax [°], the torsional stiffness [Nm/°] as the linear part of the load/deformation curve, and the energy to failure [Nm rad].

### Musculoskeletal lower limb model

The previously published musculoskeletal model of the lower limb in sheep was used to calculate the acting torsional moment along the metacarpal bone axis within a complete gait cycle [21]. The moment (Mz) was calculated as 0.0027 body weight per meter (BWm). Expecting the body weight of a black-face sheep to be approximately 85 kg a maximum torsional moment of 2.25 Nm would act on the metacarpal bone during a regular gait cycle. Assuming a safety factor of three, the metacarpal and metatarsal bones would have to withstand a torsional moment of 6.75 Nm during a normal gait cycle.

### Animals

Thirteen adult, female, black-face sheep (mean weight 66 kg; standard deviation ± 5 kg; age ≥ 2.5 years) were included in the study. All animals received 16 drill holes in total. With a diameter of 6 mm and a depth of 15 mm eight drill holes were performed in the proximal and distal epimetaphyseal bone of the femur and humerus bilaterally. Additionally two diaphyseal, unicortical drill holes (6 mm Ø) were placed in the proximal-lateral and distal-medial diaphysis of the metacarpus (MC) and metatarsus (MT) bilaterally. The drill hole defects in the right humerus, femur, metacarpus and metatarsus were placed in one surgery and defect healing was evaluated nine weeks post-surgery. Surgery on the left side was performed six weeks later. Thus, the samples of the left side were evaluated after a healing time of three weeks. All samples were harvested after sacrifice for the purpose of showing an early stage of healing after three weeks (left humerus, femur, metacarpus, metatarsus) or a later healing time point nine weeks after surgery (right humerus, femur, metacarpus, metatarsus) (Fig. 1). Drill holes were left empty, filled with different soft, non-load bearing scaffolds, and bone replacement materials. Additionally, some defects in eight of the twelve sheep were filled with an autologous, cancellous bone graft harvested from the right and the left iliac crest. Cancellous bone graft was used as a positive control in the evaluation of the different filling materials. The empty drill holes were used as a negative control. All animal experiments were conducted following national and international regulations for the care and use of laboratory animals and were approved by local authorities (approval number G0341/12, LaGeSo, Berlin, Germany).

### Surgical procedure

**Fig. 2:**
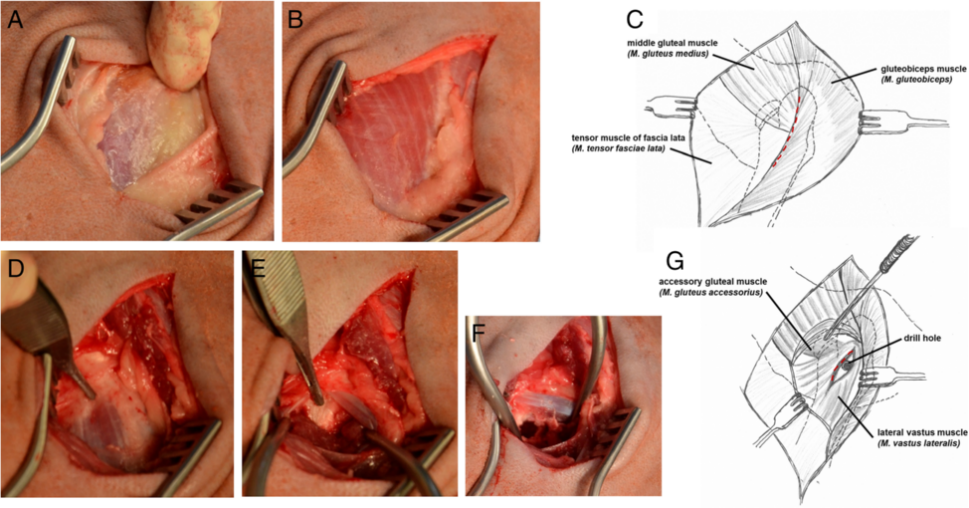
Surgical approach to the epimetaphyseal drill hole defect of the proximal femur (**a-g**). The cut through the skin exposed the underlying middle gluteal muscle, gluteobiceps muscle and the tensor muscle of the fascia lata (**a-c**). The drill hole was placed 2.5 cm distal to the tip of the great trochanter (blackface sheep) at the origin of the lateral vastus muscle and just distal to the tendon of the accessory gluteal muscle (**d-g**)

Animals were kept at least one week prior to surgery in the stable of the Charité-Universitätsmedizin Berlin. Food was withdrawn 12–16 h before surgery and water was available ad libitum at all times. Anesthesia was induced by intravenous administration of 0.5 – 1.5 mg thiopental-natrium (Trapanal®, Altana Pharma GmbH, Konstanz, Germany). Surgeries were performed under balanced anaesthesia after endotracheal intubation with a 9 mm tube. For maintenance of anesthesia a mixture of anesthesia gas (1.8–2.0 % isoflurane, 20–30 % nitrous oxid, pure oxygen) were delivered and fentanyl dihydrogen citrate in a dosage of 0.2 mg per kg body mass as intravenously bolus injections for analgesia. 3 g amoxicillin (Unacid®, Pfizer Pharma GmbH, Karlsruhe, Germany), 500 mg metronidazol (Metronidazol infusion solution, Fresenius Kabi GmbH, Bad Homburg, Germany) were given intravenously as antibiotic and 120 mg pantoprazole (Actavis GmBH, Muenich-Riem, Germany) for protection of the abomasum. During anesthesia 500 ml of a plasma expander (Vololyte 6 %, Fresenius Kabi GmbH, Bad Homburg, Germany) and 3000 ml of an electrolyte solution (Sterofundin, B.Braun Melsungen AG, Melsungen, Germany) were given for stabilisation of the cardio-vascular system. The skin was prepared for surgery by clipping, washing and shaving. Disinfection of the surgical field was performed with iodine (Braunoderm®, B.Braun Melsungen AG, Melsungen, Germany) followed by sterile draping. For all approaches a Weitlaner and a Gelpi retractor were used (Fig. 2). Bleeding was stopped with cautery.

#### Epimetaphyseal approaches

##### Proximal femur

**Fig. 3:**
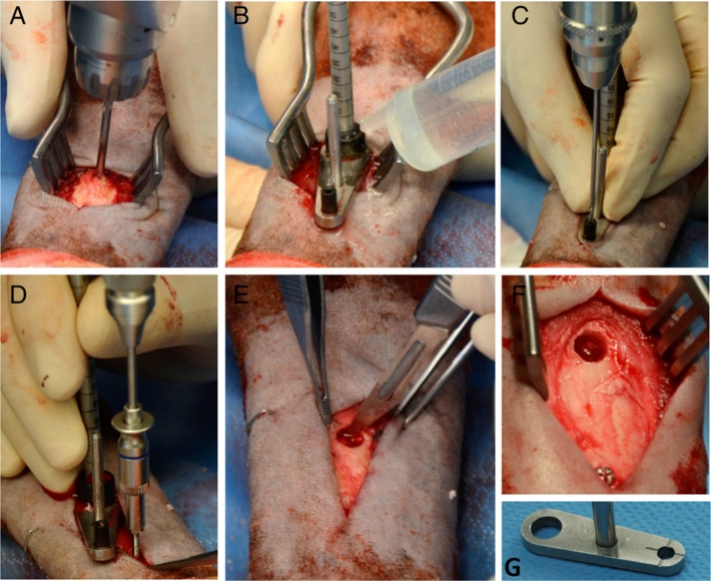
Surgical procedure and custom made devices used for preparation of a diaphyseal drill hole defect (**a-g**). **a** Positioning of the k-wire. **b** The cannulated headed reamer is used to drill the 6 mm unicortical hole. **c** The custom-made drilling template is used to position the second 1.5 mm drill hole for the 2 mm screw in a distance of exactly 2 cm adjacent to the defect. **d** With the screw holder the self-tapping screw was inserted. **e** The hole was cleaned of any periosteum and flushed with saline solution. **f-g** The cruciform slots in the screw head and the marks on the harvesting template were aligned for easy sample harvesting at the time of necropsy

With the animal positioned in underlying lateral recumbency, the femur was rotated internally. The skin over the palpable trochanter major of the femur was incised longitudinally, approximately 5 cm along the proximal part of the femur (Fig. 2a). The fascia lata was incised longitudinally, allowing the superficial gluteal and biceps femoris muscle to be retracted caudally. The middle gluteal muscle was mobilized with scissors and then it was retracted proximally until the origin of the vastus lateralis muscle was exposed. The proximal margin of the vastus lateralis muscle was incised with a scalpel and then elevated with a raspatory to expose the underlying bone approximately 2.5 cm distal to the trochanter major (Fig. 2b-g). This standardized procedure ensures exact positioning of a kirschner wire (shown in Fig. 3a for a diaphyseal drill hole) inserted in the small region of dense trabecular bone of the epimetaphyseal region of the proximal femur distal to the trochanteric fossa which was verified by careful blunt palpation caudal to the greater trochanter. The black-faced sheep is larger than the Swiss alpine sheep, so that the positioning differs from the hitherto described procedure to create a 15 mm deep defect surrounded by cancellous bone. With a custom-made drill sleeve and a cannulated headed reamer (Arthrex GmbH, Munich, Germany) the drill hole was enlarged to 6 mm in diameter and a depth of 15 mm under constant irrigation with saline solution (shown in Fig. 3b for a diaphyseal drill hole). For subsequent harvesting of the bone surrounding the drill hole sample at necropsy a second 1.5 mm Ø drill hole was placed at a distance of exactly 2 cm towards the diaphysis of the bone by the use of the custom-made drill template (Fig. 3b). A 2 mm stainless steel self-tapping cortical bone screw, 12 mm in length (Synthes GmbH, Umkirch, Germany) was inserted as a landmark (shown in Fig. 3b-e for a diaphyseal drill hole). A second custom-made “harvesting template” with a 6 mm hole on one side and a 2 mm hole on the other side with notches, corresponding to the cross-slot of the screw, was placed over both drill holes (shown in Fig. 3f-g for a diaphyseal drill hole). By the use of a screw driver, the cross-slot of the 2.0 mm screw was orientated so that it corresponded to the notch of the harvesting template. The drill hole were carefully flushed with saline solution to eliminate bone debris and dried with sterile compresses before filling with different soft bone graft substitutes. The vastus lateralis, gluteal and biceps femoris muscles and the fascia lata (ETHICON Vicryl 2–0, Johnson & Johnson Medical GmbH, Norderstedt, Germany), and subcutaneous tissue (ETHICON Vicryl 3–0, Johnson & Johnson Medical GmbH, Norderstedt, Germany) were closed with continuous sutures. The skin was closed separately with simple interrupted sutures of non-absorbable material (ETHICON Prolene 3–0 Johnson & Johnson Medical GmbH, Norderstedt, Germany). Colloidal aluminium spray was used for wound covering.

##### Proximal humerus

With the animal positioned in lateral recumbency, the humerus was stretched and rotated externally. The skin incision began at the level of the greater tubercule following the curve of the humerus approximately seven cm distal. After dissecting the subcutaneous fascia the cranial border of the acromial part of the deltoid muscle was elevated. The k-wire was positioned 2.5 cm distal to the edge of the tuberculum majus and at a distance of 2 cm lateral to the crista tuberculi majoris and drilled in a medial orientation to the caput humeri in all animals. Due to this standardized procedure the exact location of the drill hole in the small region of dense trabecular bone of the epimetaphysis of the proximal humerus was guaranteed. The drill hole was performed as described for the femur. A screw was placed as a landmark at a distance of two cm towards the shaft region of the humerus as described for the femur. The surgical approach was closed by continuous sutures in the detoideus muscle and subcutaneous tissue, and with simple interrupted sutures in the skin.

##### Distal humerus

With the sheep lying in lateral recumbency, a skin incision was made over the lateral epicondyle of the humerus. The subcutaneous and deep fasciae along the cranial margin of the lateral head of the triceps muscle were incised and the lateral collateral ligament was identified on the lateral epicondyle. The drill hole was placed exactly in the center of the lateral epicondylus by the same procedure as used for the proximal femur. A screw was placed as a landmark in a distance of two cm towards the shaft region of the humerus. Both fasciae, the lateral collateral ligament, and the subcutaneous tissue were sutured continuously, while the skin was closed with simple interrupted sutures.

##### Distal femur

The surgical approach to the distal femur was performed from the medial side and therefore the animal had to be repositioned into dorsal recumbency, after all drill holes with a lateral approaches had been performed. The patella, tibia plateau and medial femur condyle are palpable. Skin incision was performed longitudinally following the curve of the femur between this landmarks. The underlying fascia was dissected and the cranial margin of the cranial belly of the sartorius muscle was incised with a scissor and cautery and retracted caudally. Then the caudal margin of the vastus medialis muscle was elevated to expose the underlying bone of the distal epimetaphyseal region. The bone surface was freed from periosteum with the raspatorium. The drill hole was placed proximal to the insertion of the medial collateral ligament. The small screw was inserted 2 cm proximal to the drill hole in direction to the shaft, as described for the other drill holes. The sartorius muscle and the fascia were closed, followed by the subcutaneous and skin suture.

#### Diaphyseal approaches

##### Proximal-lateral and distal-medial metacarpal and metatarsal bones

The diaphyseal unicortical drill holes were placed in the proximal and distal parts of the left and right metacarpal and metatarsal bone. The proximal drill holes were placed from lateral, whereas the distal drill holes were performed from medial after repositioning of the sheep to the reverse side. The drill holes were placed exactly at the border of the first to second proximal quarter and the third and fourth quarter of the metacarpus and metatarsus. For a standardized positioning, the bone length was determined with a fluoroscope before sterile coverage of the sheep and the exact positions of the drill holes were marked with a surgical staple in the skin.

A 2 cm longitudinal incision was made through the skin, subcutaneous tissue and periosteum to the bone, taking care to avoid the digital extensor tendons. The k-wire was placed in the center of the bone and drilled transversely through one cortex (Fig. 3a). The unicortical drill hole was enlarged to 6 mm using the same procedure as for the epimetaphyseal bones (Fig. 3b). The second smaller drill hole for the locating screw was placed towards the shaft (Fig. 3c,d,f). The drill hole was cleaned of any periosteum, flushed with saline solution and dried with sterile gauze sponges (Fig. 3c). After defect filling, a thin layer of connective tissue and the subcutaneous skin were closed over the drill defect with continuous, absorbable sutures. The skin was closed with simple interrupted sutures of non-absorbable suture material.

### Animal care

The sheep were observed untill their recovery from general anesthesia. Immediately after surgery they were housed in small groups without restriction to movement. Clinical examination was performed daily. Postoperatively the sheep received a 75 μg/h fentanyl patch (Durogesic® 75 μg/h, Jannsen-Cilag GmbH, Neuss, Germany), which was renewed after three days and a dose of 2.2 mg flunixin-meglumin (Finandyne®, Intervet Deutschland GmbH, Unterschleißheim, Germany) per kg body mass daily for the first seven days after surgery. First day after surgery the sheep received additionally 500 mg metronidazol and 120 mg pantoprazole intravenously. 8.000 IE Dihydrostreptomycinsulfat, 4.8 mg Benzylpenicillin- Procain, and 3 mg Benzylpenicillin-Benzathin (Veracin®, Albrecht GmbH, Aulendorf, Germany) per kg body weight were given every second day until the skin sutures were removed.

### Radiographic analysis

**Fig. 4:**
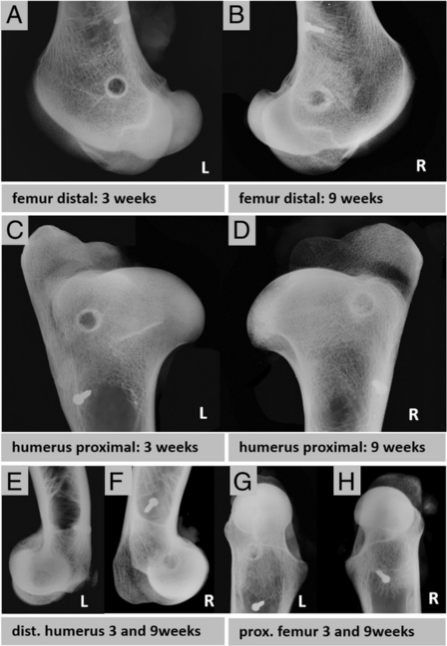
Radiographic faxitron images (**a-h**). **a-b** Epi-metaphyseal drill hole defects in the left and right distal femur, **c-d** proximal humerus, **e-f** distal humerus, **g-h** and proximal femur are shown after 3 and 9 weeks observation time. The 2 mm small screw marks the position of the drill hole in a distance of 2 cm towards the shaft region. For an exact sample harvesting the custom made harvesting template was used

**Fig. 5:**
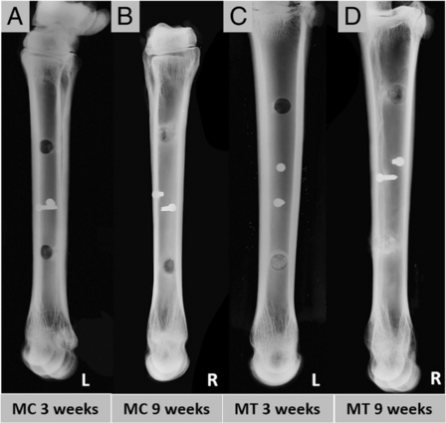
Radiographic faxitron images of the diaphyseal drill hole defects in the left and right metacarpal and metatarsal bone (**a-d**). Defects are located at the interface of the second to third quarter of each bone. For the exact sample harvesting after the observation time of either 3 or 9 weeks a 2 mm screw was placed in a distance of 2 cm to the drill hole (**a-d**). Image **c** shows the left MT bone in which the proximal drill hole was left empty and the distal drill hole was filled with autologous cancellous bone graft 3 weeks after surgery. 9 weeks after surgery the distal defect was hardly visible (autologous cancellous bone graft) (**d**) whereas the proximal empty control defect is still remarkable (**c-d**)

Standardized latero-medial radiographs were performed immediately after each surgery and microradiographed (Faxitron, Bioptics, Arizona, USA) ex vivo after harvesting of the femurs, humeri, metacarpi, and metatarsi (Figs. 4a-h and 5a-d).

### Statistical methods

All statistical analyses were performed using SPSS 20.0 Programm SPSS® Version 20 (SPSS GmbH Software, München, Germany). A p-value of <0.05 was considered as significant. A univariate ANOVA was performed. Data are presented in box plots showing the median, 25 and 75 percentile and min–max (whiskers).

## Results

### Biomechanical in vitro testing

Biomechanical torsional test to failure was performed in order to investigate the stability of the bones after placing the diaphyseal drill holes and to ensure that the normal physiological strains do not affect the bones to a point of failure.

#### Comparison native MC vs. native MT bones

**Fig. 6:**
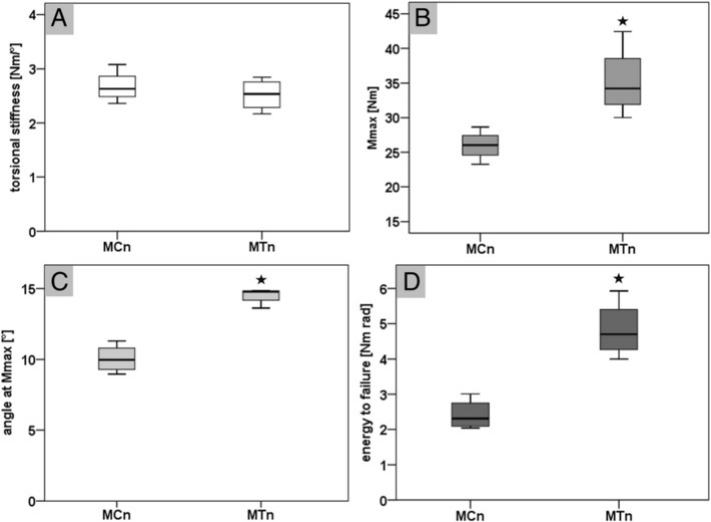
Biomechanical torsional test results for the native metacarpal (MCn) and metatarsal (MTn) bones (**a-d**). **a** shows the results for the torsional stiffness in Nm/° without a statistical significant difference beween both bone types. **b** shows the maximum torsional moment until failure (Mmax in Nm) with a significant higher value for the metatarsal bones (*p* = 0.017). **c** shows the results for the angle at Mmax in ° with a significant larger angle for the native MT bones (*p* < 0.001). **d** shows a significant higher energy to failure (in Nm rad) for the native MT bones compared to the native MC bones (*p* = 0.002)

The comparison of the biomechanical torsion test result of the native MC (MCn) and MT (MTn) bones showed no significant difference in their torsional stiffness (Fig. 6a). The native MT bones had in comparison to the MC bones a statistically significant higher maximum torsional moment until failure (*p* = 0,017), angle at Mmax (*p* < 0,001), and energy to failure (*p* = 0,002) (Fig. 6b,c,d).

#### Comparison native vs. drilled MC bones

**Fig. 7:**
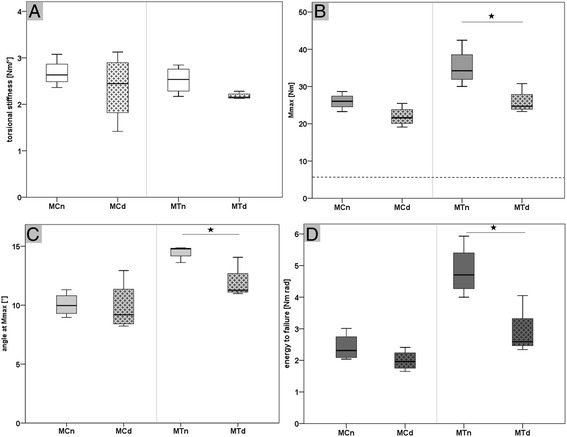
Biomechanical torsional test to failure results of native and drilled metacarpal and metatarsal bones (**a-d**). **a** shows the torsional stiffness of the native and drilled metacarpal (MCn vs. MCd) and metatarsal (MTn vs. MTd) bones without a statistical significant difference. **b** shows the result of the maximum torsional moment until failure (Mmax) for the native and drilled metacarpal and metatarsal bones. The dashed line refers to the calculated maximal torsion moment of 6.75 Nm acting during a normal gait cycle (moments gait cycle = 0.0027 body weight per meter × 85 kg body weight sheep x safety factor 3). The Mmax for the drilled MT bones was significantly lower (*p* = 0.023) in comparison to the native MT bones, but within the same range as the native and drilled MC bones. **c** shows a statistical significant lower angle at Mmax (*p* = 0.016) for the drilled MT bones in comparison to the native MT bones. **d** shows a significant lower energy to failure (*p* = 0.014) of the drilled MT bones in comparison to the native MT bones

The comparison of the native MC and the drilled MC (MCd) bones showed no statistical significant differences in all measured parameters (Fig. 7a-d). The two drill holes reduced the mean torsional stiffness by 13 %, the mean Mmax by 16 %, the mean angle at Mmax by 1 %, and the mean energy until failure by 15 %.

#### Comparison native vs. drilled MT bones

The comparison of the native MT and drilled MT (MTd) bones showed no statistical significant difference in torsional stiffness (Fig. 7a). The drilled MT bone showed a statistical significant decreased of the maximum torsional moment (*p* = 0.023), angle at Mmax (*p* = 0.016), and energy to failure (*p* = 0.014) (Fig. 7b-d). The two drill holes reduced the mean torsional stiffness by 13 %, the Mmax by 26 %, the mean angle at Mmax by 18 %, and the mean energy until failure by 39 %.

#### Comparison drilled MC vs. drilled MT bones

The comparison of the drilled MC and MT bones showed no statistical significant differences in all parameters (Fig. 7a-d).

In summary, the native MC and MT bones showed no statistical significant difference in torsional stiffness but in all other measured parameters (Fig. 6a-d). Prepared with two 6 mm drill holes, only the bones of the hind legs (MT) showed a statistical significant decrease of the maximum torsional moment, angle at Mmax, and energy to failure, ranging in the same value as the bones of the front legs (MC; Fig. 7a-d). Between the drilled bones of the front and hind legs no statistical significant difference could be observed in any measured parameter.

### In vivo results

All 13 sheep recovered from surgery and anesthesia without any complications. One animal had to be sacrificed after seven weeks due to a fracture of the left metacarpus one week after the second surgery. The other twelve animals survived the observation time of nine weeks without any complications. The general condition and physiological behavior was not affected by the surgery, so they were left in their group at all times. Only a minimal mean weight loss of 3 % and 0.5 % after the first and second surgery was registered over the course of time. All animals showed a declining moderate to slight degree of lameness in the limbs undergoing surgery until day seven post-surgery. The additional harvesting of cancellous bone graft from each iliac crest did not appear to have any impact on the severity of lameness. One animal developed a severe lameness of the left forelimb between day 10 to 21 post surgery, which was treated with a daily dose of 2.2 mg flunixin-meglumin (Finadyne®, Intervet Deutschland GmbH, Unterschleißheim, Germany), 8.000 IE Dihydrostreptomycinsulfat, 4.8 mg Benzylpenicillin- Procain, and 3 mg Benzylpenicillin-Benzathin (Veracin®, Albrecht GmbH, Aulendorf, Germany) per kg body weight every second day until recovery.

### Radiographic analysis

Radiographic analysis was performed for the documentation of the surgery result, as a support for sample explantation and a broad characterization of the healing result after 3 or 9 weeks of healing.

Epimetaphyseal drill holes of proximal and distal femur and humerus were in general more difficult to detect on in the radiographic images than diaphyseal drill holes, due to the surrounding trabecular bone. Three weeks after surgery the drill hole defects showed a slightly more radiodense zone surrounding the defect and mineralized callus formation from the edges towards the center of the drill hole (Fig. 4a,c,e,g). After nine weeks the sourrounding radiodense zone enlarged and the mineralized callus formation within the defect had progressed (Fig. 4b,d,f,h).

The drill holes of the left metacarpus and metatarsus were clearly detectible on the micro-radiographic images three weeks after surgery. The cortical boundary was sharp (Fig. 5a,c). Whereas the diaphyseal drill holes of the right metacarpus and metatarsus showed an advanced healing result with a strong bony mineralized callus formation from the cortex towards the center of the drill hole nine weeks after surgery (Fig. 5b,c).

The results indicate an advanced healing result for the diaphyseal drill holes after 9 weeks of healing and in general the difficulty to detect epimetaphyseal drill holes after a healing time of 9 weeks and the necessity of a reference screw.

## Discussion

With this study we introduce a new large animal model for the investigation of epimetaphyseal and diaphyseal drill hole defect regeneration in sheep. To the authors knowledge, a preclinical large animal model in which cancellous and cortical defect healing involving intramembraneous ossification can be investigated has not yet been published. Optionally two different healing time points can be analyzed in the same animal. There are several small and large animal osteotomy models for the investigation of a critical or non-critical sized cortical bone defects established, which involve in uncritical defect sizes the process of endochondral ossification [2, 3, 14, 22]. For the investigation of the special healing pattern of epimetaphyseal defects, independent small and large animal osteotomy models are also established [6–8, 10–13, 23–25]. In these models, cancellous bone defects regenerate under stable biomechanical conditions directly through intramembraneous ossification almost without intermediary cartilage [6–8, 10, 11, 13, 23]. In comparison to these models with only one harvested sample per animal, drill hole models have the advantage of an easy set-up. They offer a good reproducibility, a low morbidity in comparison to critical size defect models, contain mostly higher sample numbers per animal, and are accepted by some authors as a healing model for a fracture gap with a stable fixation [1, 26]. There is only one small animal drill hole defect model from Monfoulet et al., which allows the investigation of cortical and cancellous bone regeneration but compared to a large animal model with small defect sizes of 1 mm in the mice femur [26].

The cancellous bone defects in the here presented model were performed in reference to the well-established drill hole defect models in epimetaphyseal bone of the proximal and distal part of the femur and humerus, albeit with some modifications and improvements [15–18]. The described surgery technique has been refined by the use of a k-wire guided drilling. Precise drilling of holes with the cannulated-headed reamer prevents a slippage of the large 6 mm drill and can avoid complications like fractures related to a failures during defect preparation as it has been described in the hitherto models [17]. Additionally, for the precise explantation of samples a method for marking was introduced by the use of two custom-made templates and a small reference screw.

The position of the drill hole in the proximal femur was adapted to the dimensions of the larger black-faced sheep compared to the hitherto used Swiss alpine sheep. To ensure a precise placement in the rather small proximal, cancellous, epimetaphyseal end of the femur, the drill hole was placed in a distance of 2.5 cm from the distal tip of the major trochanter. Other drill hole defect models use the proximal epimetaphyseal part of the tibia, but due to the short area with stable cancellous bone, the tibia was not included in this model [27–29]. In addition, the approach to the distal femur was modified in our model and performed from the medial side to avoid the more pronounced muscle layer on the lateral side of the condyles.

For the investigation of diaphyseal drill hole defect regeneration, the metacarpal and metatarsal bones were chosen, according to their high similarity in shape and size and their general acceptance in cortical defect models [1]. Petrizzi et al. used the metacarpal bone of the left side for a bicortical drill hole model for the investigation of the effect of naloxone on bone regeneration but without a biomechanical test of the metacarpal bone to evaluate the influence of the defect size on the bone [30]. Other studies in sheep used the tibia for the placement of smaller single or multiple, uni- or bicortical drill hole defects [31, 32].

For our model, both unicortical defects per bone were placed in a maximal distance to each other in the proximal lateral and distal medial shaft region to have low mutually influential effects. The drill hole defect size was determined to 6 mm in diameter, to stay below the recommended defect sized of 50 % related to the diameter of the utilized metacarpal and metatarsal bones [20]. The epimetaphyseal drill hole defect size was adapted to the diaphyseal drill hole size. Prior to the in vivo testing, a biomechanical torsion test until failure of native and drilled metacarpal and metatarsal bones of four test sheep were performed to ensure that our defect does not affect the bone stability in a way that would induce fractures during physiological load. The native MC bones of the front legs showed a significant lower maximum torsion moment until failure in comparison to the MT bones, as they are macroscopically slightly smaller and thinner. Nevertheless, defect drilling significantly decreased only the Mmax, angle at Mmax, and energy to failure of the MT bones compared to the corresponding healthy contralateral MT bones. The torsional stiffness, Mmax, angle at Mmax, and energy to failure of the drilled MT bones ranged after defect creation at the same level as the healthy and also the drilled MC bones. Therefore we concluded, that MC bones of the front legs and MT bones of the hind legs prepared with two 6 mm unicortical drill holes might be comparable in their biomechanical behavior. Biomechanical properties of the MT bones of the hind legs were more affected by the drill holes than the MC bones of the front legs. Influence of slightly different loading situation on defect healing might be normalized by a distribution of samples in a randomized complete block design, leading to an equalized number of samples from one material in the MC or MT bones. The calculated existing moments on the MT sheep bone in the musculoskeletal lower limb model with a high safety margin, were much lower during a normal gait cycle than the maximum torsion moments until failure. Therefore, we concluded that MC and MT bones prepared with two 6 mm unicortical drill holes might withstand the acting moments during healing time. Nevertheless, a failure cannot be completely excluded because one animal had to be sacrificed earlier due to a spiral fracture of the left MC bone. This shows that the existing peak moments in an experimental sheep stable without restriction to movement might range up to the level of the measured moments until failure. However, a failure rate of approximately 8 % for the diaphyseal drill holes seems to be tolerable, compared to the amount of 96 epimetaphyseal and 96 diaphyseal evaluable samples within 12 sheep, 8 of them with additional cancellous bone graft harvesting from the iliac crest. Particularly, as the sheep health status and behavior was only slightly affected during the study.

In our opinion, the combination of calculation of acting moments from the musculoskeletal sheep model and a previous biomechanical failure test proved to be an adequate technique for the estimation of acting forces and safety against failure of this new in vivo defect model. Investigations of promising biomaterials in this model focus on the stimulation and improvement of intramembranous bone healing under stable biomechanical conditions, but provide the possibility of a regular animal housing without restriction to movement like cast bandage or sling-system immobilization.

**Fig. 8:**
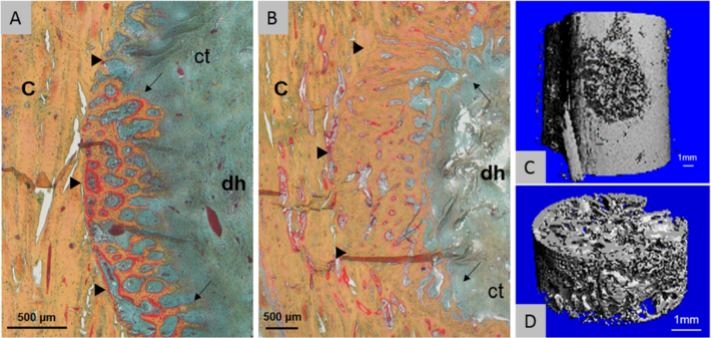
Histology and micro computer tomography of diaphyseal drill holes (**a-d**). **a** Movat’s pentachrome stained drill hole defect (dh) three weeks after surgery (*left metatarsal bone*) shows woven bone formation via direct intramembranous ossification (*arrows*; osteoid stained in *red*; ct = connective tissue). The border between the old cortical bone (**c**) and the newly formed bone is clearly distinguishable (*triangles*). B After nine weeks, bone defect healing progressed (*arrows*), but the interface between newly formed bone and old bone is still noticeable (*triangles*). **c-d** micro CT image (vivaCT 40, Scanco Medical AG, Brüttisellen, Switzerland; 17,5 μm voxel size) of a metacarpal drill hole defect three weeks after surgery filled with autologous cancellous bone graft. **d** The mineralized tissue within the drill hole defect can be analyzed separately

Besides the performed radiographic follow-up images, detailed defect healing could potentially be evaluated *in vivo* with a high-resolution peripheral quantitative computer tomography (HR-pQCT, Scanco Medical AG, Brüttisellen, Switzerland) in the diaphyseal regions. After sacrifice, a variety of analysis methods are feasible as the histological evaluation (Fig. 8a-b) or a micro computer tomography (μCT) analysis (Fig. 8c-d) to investigate the healing outcome.

Nevertheless, this model is limited to the certain drill hole defect size and localizations and cannot replace critical-size defect studies or the investigation of biomaterials in the later targeted application site. Besides, sheep have cancellous and cortical bone and show a similar bone remodeling and turnover rate as humans, but have a plexiform bone structure with few Haversian canals unlike the human bone [33, 34]. As no single animal model completely recapitulates human bone regeneration each species provides advantages and limitations [35, 36], which need to be considered carefully before choosing the suitable animal for a drill hole model. In general, an extend of this drill hole model to other large animals as goats or pigs is conceivable. As goats have a comparable body weight and metabolic rate to humans [36, 37], they are commonly used as a model in bone defect [38, 39] and osteochondral defect studies [39, 40]. The huge weight of adult pigs with proportional short limbs could be challenging within this model [41], albeit mini-pigs might be suitable as they have been already used in studies to investigate osteointegration and bone defect healing [42, 43]. Nevertheless, the switch to another species would require further biomechanical evaluations for a feasible defect size. Additionally, it would be necessary to adapt the surgical approach as well as the observation time points to the respective animal species.

## Conclusion

This new preclinical large animal drill hole model allows the simultaneous investigation of new bone tissue engineering strategies in cancellous and cortical bone defect healing sites. The sheep model combines the advantages of an adequate defect size for the targeted application in a patient and a sufficient number of samples per animal for the investigation of intramembranous bone defect regeneration. Beside that the combination of the calculation of acting moments from the musculoskeletal lower limb model and biomechanical in vitro testing proofed to be an adequate method to keep the accidental fracture risk low. This model is intended to be used as a simplified screening tool for the selection of promising biomaterials before an application in a complex treatment site.
